# Definition of the binding specificity of the T7 bacteriophage primase by analysis of a protein binding microarray using a thermodynamic model

**DOI:** 10.1093/nar/gkae215

**Published:** 2024-04-10

**Authors:** Georg Lipps

**Affiliations:** Institute of Chemistry and Bioanalytics, University of Applied Sciences Northwestern Switzerland, 4132 Muttenz, Switzerland

## Abstract

Protein binding microarrays (PBM), SELEX, RNAcompete and chromatin-immunoprecipitation have been intensively used to determine the specificity of nucleic acid binding proteins. While the specificity of proteins with pronounced sequence specificity is straightforward, the determination of the sequence specificity of proteins of modest sequence specificity is more difficult. In this work, an explorative data analysis workflow for nucleic acid binding data was developed that can be used by scientists that want to analyse their binding data. The workflow is based on a regressor realized in scikit-learn, the major machine learning module for the scripting language Python. The regressor is built on a thermodynamic model of nucleic acid binding and describes the sequence specificity with base- and position-specific energies. The regressor was used to determine the binding specificity of the T7 primase. For this, we reanalysed the binding data of the T7 primase obtained with a custom PBM. The binding specificity of the T7 primase agrees with the priming specificity (5′-GTC) and the template (5′-GGGTC) for the preferentially synthesized tetraribonucleotide primer (5′-pppACCC) but is more relaxed. The dominant contribution of two positions in the motif can be explained by the involvement of the initiating and elongating nucleotides for template binding.

## Graphical Abstract

**Graphical Abstract:**
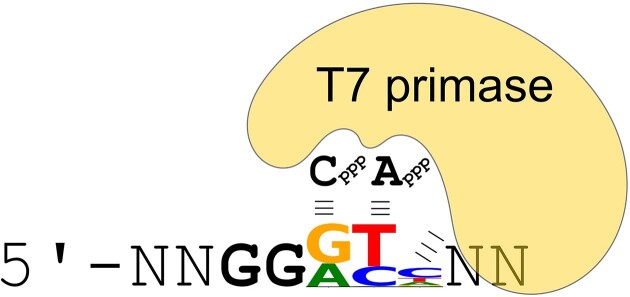

## Introduction

The regulation of transcription and translation is mainly realized by proteins, which bind sequence-specifically to nucleic acids. Some proteins have acquired exquisite specificity and bind with high affinity to selected DNA or RNA sequences. The systematic analysis of the specificities of transcription factors became possible with the development of PBM which incorporated in ∼42 000 double-stranded DNA probes the occurrences of all possible 10mers (1,2). The transcription factors are fluorescently labelled and incubated with the microarray, washed intensively, and the remaining fluorescence signal of the spots is used as proxy for the binding of the transcription factor to the probe sequence. The binding data can then be analysed with algorithms that are based on motif enrichment or with thermodynamic models, as reviewed in Weirauch *et al.* (3). Besides PBM, there are alternative experimental strategies to investigate the specificity of DNA-binding proteins with the most prominent being SELEX, RNAcompete and Chromatin-Immunoprecipitation (4).

Different algorithms for defining the sequence specificity have been developed. Some more advanced data analysis procedures incorporate additional properties for predicting the protein binding to a DNA sequence, such as DNA shape (5,6) and the effect of neighbouring bases within a motif and the positional bias of PBMs (7,8). However, for most transcription factors, the predictions improve not or only slightly with these additional properties. Rather, the independent contribution of each position in the motif remains to be the major determinant of specificity of DNA binding proteins (9). The independent contribution of each position of the motif reinforces that the prominent visualisation of the motif as an information logo is appropriate, since logo presentation cannot capture DNA shape or correlations between positions.

For RNA binding proteins, an alternative approach to systematically assess specificity has been developed (10,11). In RNAcompete the GST-tagged RNA-binding proteins are incubated with an excess of around 250 000 different single-stranded RNA probes. The RNAs bound to the RNA-binding protein are then separated from the RNA pool by a GST pulldown. Next, the bound RNA is eluted, labelled and its identity revealed by hybridisation with a microarray. Single-stranded RNA can adopt different conformations and the binding of the RNA is influenced by sequence and RNA structure. The former can be quite easily investigated with the methods developed for PBM. In contrast, the physiologically relevant native structure of the RNA might not form as the RNA probes are quite short (30–40 nucleotides). More advanced algorithms, e.g. BEAM, however predict RNA binding by taking account the sequence and the structure of the RNA (12).

PBMs, SELEX and RNAcompete are successful for analysing nucleic acid binding proteins with prominent specificities. In these cases, the protein will bind strongly to only a minor subset of the probes. More difficult is the analysis of proteins which have only a limited sequence specificity. In these cases, the proteins will bind to many probes or may even bind multiple times to a single probe.

Bacterial primases display a certain sequence specificity (13). They usually only prime in the context of a short 3 bases long motif. In general, the priming specificity of the primases is not very well investigated. Nevertheless, priming specificity is known for the best-investigated bacterial primases. For example, the *Escherichia coli* primase primes at 5′-CTG (14), the *Staphylococcus* primase prefers 5′-CTA (15) and the primase from the Bacteriphage T7 starts primers at the motif 5′-GTC (16). These trinucleotide recognition sequences are only partly used as template. In case of the T7 primase the 3′ nucleotide cytosine is cryptic and the primer synthesis starts at the central base generating the initial dinucleotide primer pppAC. The priming specificity of the primases might help to coordinate the DNA synthesis at the lagging and leading strand of the bacterial replisome. For the T7 replisome it could be demonstrated by single-molecule studies that upon primer synthesis at the lagging strand the DNA replication is paused at the leading strand (17). In the context of the replisome, T7 preferentially synthesizes a tetraribonucleotide of pppACCC, pppACCA and pppACAC. These short RNA primers are efficiently synthesized before being released and handed-off in a time-limited step towards the T7 DNA polymerase which then proceeds with DNA extension of the Okazaki fragment (18).

In a recent work Soffer et al. investigated the primase sequence specificity of the bacteriophage T7 primase with a custom made PBM (19,20). The custom PBM had the known T7 priming site (5′-GTC) in the centre and was flanked by variable sequences composed of 2–3 nucleotides. For the binding experiment, a his-tagged T7 primase was incubated with the PBM and excess protein was washed away. Next, the T7 primase binding was detected with a fluorescent antibody against the his-tag. The median intensities of ∼3000 single-stranded DNA probes were normalized to a binding score (scale of 0 to 1). The obtained binding data was then analysed by machine learning technique. The authors established a linear model which used as features the k-mers counts of the probe sequences. The mean absolute error of the model was around 0.1 (∼10% of the binding score range). This model, however, did not allow to further describe the specificity of the T7 primase in terms of a classical sequence motif. The specificity of the primase is in fact ‘hidden’ in the *k*-mers coefficient of the model. The result of this particular machine learning exercise points towards a general problem in machine learning, which is the lack of explainability and the high complexity of the obtained models.

Given the robust body of evidence that DNA binding specificity can be determined using the classic approaches developed for the analysis of PBM, Selex and RNAcompete the data of Soffer et al. was reanalyzed with a strictly thermodynamic model of protein binding. For this means a regressor for motif discovery was developed within the scikit-learn environment and incorporated into a Jupyter notebook to allow for facile and flexible explanatory analysis of nucleic acid binding data.

## Materials and methods

### The regressor

The regressor was realized as a scikit-learn regressor, which allows to use the powerful framework of the scikit-learn module for later analysis.

The regressor requires as input the probe sequences and the associated binding data of each probe. In its current implementation, it is required that there is a linear relationship between the protein occupancy on the probe and the binding data used as input for the regressor. This prerequisite shall be met in a well-performed experiment with experimental conditions avoiding probe saturation.

Beside the input data, the regressor requires as a parameter the motif length. Depending on the motif length, a list of subsequences with the same motif length are constructed from the input probe sequence. The subsequences, which are the discrete potential binding sites, are taken along the whole input sequence and differ in the starting position by one nucleotide.

To predict the binding of a protein to a nucleic acid sequence, a thermodynamic model similar to the one developed by Stormo (21) and later refined by Morozov (8) was used. Briefly, the occupancy for each subsequence was calculated as follows:


\begin{eqnarray*}occupanc{{y}_{subsequence}} = \frac{{\left[ {protein} \right]}}{{\left[ {protein} \right] + {{K}_D}}}\end{eqnarray*}

With [protein] the protein concentration during the incubation with the probes and *K*_D_ the dissociation constant between the protein and the subsequence. In case the protein concentration is assumed to be low compared to the dissociation constant *K*_D_ the equation above can be simplified by omitting the protein concentration in the denominator. This approximation was realised in MatrixReduce (22) but was not consider here.

The dissociation constant itself can be calculated as follows:


\begin{eqnarray*}{{K}_D} = \ {{e}^{\frac{{\Delta {{G}^0}}}{{RT}}}}\end{eqnarray*}

With $\Delta {{G}^0}$ the standard free energy of the binding (association) reaction between protein and subsequence.

The Standard Gibbs energy of the association reaction can now be expressed as the sum over all positions of the subsequence with each nucleotide having a distinct position dependant free energy of binding:


\begin{eqnarray*}\Delta {{G}^0} = \mathop \sum \limits_{positions,\ nucleotides}^{} {{s}_{position,\ nucleotide}}\ \Delta {{G}_{position,\ nucleotide}}\end{eqnarray*}

$\Delta {{G}_{position,\ nucleotide}}$ represents the free energy matrix of the size 4*motif length and ${{s}_{position,\ nucleotide}}$ encodes the subsequence. While such an energy matrix is formally correct, it is not suitable for optimisation during motif finding since the binding experiments only yield information about the specificity of binding but not the affinity (that would require the absolute occupancy of the probes, which is usually not known). Therefore, the expression of $\Delta {{G}^0}$ is reformulated as follows:


\begin{eqnarray*}&& \Delta {{G}^0} = \ \Delta {{G}_0} \nonumber\\ &&+ \mathop \sum \limits_{positions,\ nucleotides}^{} {{s}_{position,\ nucleotide}}\ \Delta \Delta G_{nucleotide,\ position}^{normalized}\end{eqnarray*}

with the condition that


\begin{eqnarray*}\mathop \sum \limits_{nucleotides}^{} \Delta \Delta G_{nucleotide,\ position}^{normalized} = 0{{\ }_{}}\ \left( {for\ all\ positions} \right)\end{eqnarray*}

The first summand, $\Delta {{G}_0}$, represents the unspecific affinity of the protein to the nucleic acid sequence of subsequence length, and the second summand describes the specificity of the protein towards subsequences with different sequences. This reformulation reduces the number of parameters to describe the specificity to three per position while $\Delta {{G}_0}$ expresses solely the affinity. $\Delta {{G}_0}$ can be estimated or fixed before optimization.

In a final step, the occupancies of all subsequences of a single probe are summed up to yield the occupancy on the probe:


\begin{eqnarray*}occupanc{{y}_{probe}} = \mathop \sum \limits_{subsequences} occupanc{{y}_{subsequence}}\end{eqnarray*}

In a well-conducted binding experiment, the number of protein molecules per probe should be very low for most probes, and in a few cases, it might reach 1–2. The described model estimates the occupancy of each subsite independently and therefore does not prohibit overlapping binding events of two neighbouring sites, which is, however, structurally not possible. Under standard setting $\Delta {{G}_0}$ is therefore set to avoid unphysiological oversaturation of single probes. Alternatively, the problem of calculating the correct occupancy in case of overlapping binding sites can be explicitly modelled (8).

The regressor calculates for each input sequence the total occupancy according to the normalized energy matrix $\Delta \Delta G_{nucleotide,\ position}^{normalized}$. The occupancies over all probes are compared with the input binding data of all probes, and the Pearson linear regression coefficient r is maximized by the regressor during motif finding, finally yielding the energy matrix that best describes the motif. The optimized energy matrix $\ \Delta \Delta G_{nucleotide,\ position}^{normalized}$ is visualized as an energy logo and, via the calculation of the frequency matrix, also as an information logo.

The regressor takes as an additional parameter whether the probes are single-stranded or double-stranded nucleic acids. For double-stranded probes, the occupancy on both strands is summed while disallowing an occupancy above 1 for each subsequence.

In essence, the regressor is a linear model which internally calculates the binding occupancy on the complete probe sequence by taking into account the agreement of the motif (expressed as energy matrix) with all the subsequences of the probe.

The previous analysis by the group of Prof. Akabayov also employed a linear model but the inner workings of the linear regression model are completely different. From each sequence the K-mer frequency was determined for a pre-chosen K. For example, for *K* = 3 the frequency of each possible trimer (4^3^= 64) in each probe sequence was calculated. This preprocessing steps transforms the 36 nucleotides of the probe sequence into 64 features which are the 64 trimer frequencies. In a linear model, the target value (here the predicted binding score) is calculated by summing over the product of weights and features (=trimer frequencies).


\begin{eqnarray*}predicted\ binding\ score = \ \mathop \sum \limits_{} weight*features\ + intercept\ \end{eqnarray*}

The weights and the intercept are now varied to find a good agreement between the predicted binding score and the actual (measured) binding score. There are several metrices to judge the agreement, in the cited work the mean absolute error (mae) was taken. Another popular metric is the Pearson correlation coefficient.

The linear model above can be solved but the high number of parameters (weights) will probably lead to overfitting. Therefore, such a linear model shall be regularized, a technique where the magnitude of the weights is penalized. In their work Lasso (least absolute shrinkage and selection operator) also called L1 regularization was chosen. Here, the sum over all absolute values of the weights is used as penalty. With L1 regularization most weights are zeroed out in the process of regularization thus the linear model is simplified as the number of parameters is decreased. In addition, the authors intentionally forced non-negative weights and used for their final models only the 10 largest weights.

While this machine learning model is also able to predict the binding score, the model itself does not have a direct physical meaning which turns out to be problematic when the binding specificity incorporated in the model shall be understood. Continuing with the example *K* = 3: In case the binding specificity would be the unambiguous dinucleotide motif 5′-GT we would expect that the weights for trimers containing 5′-GT are positive and the weights for trimers not containing this dinucleotide would be zero or negative. For ambiguous motifs like 5′-G/A-T/C we would expect that all trimers containing either 5′-GT, 5′-GC, 5′-AT and 5′ AC have positive weights. As can be seen by these examples the decoding of the weights (which may also contain the preference for certain dinucleotide or trinucleotides) to a sequence motif with independent positions is possible but not straightforward.

### The workflow

In the standard workflow realized within a Jupyter Notebook, the input data is split into a training (80%) and test (20%) datasets.

**Figure 1.**
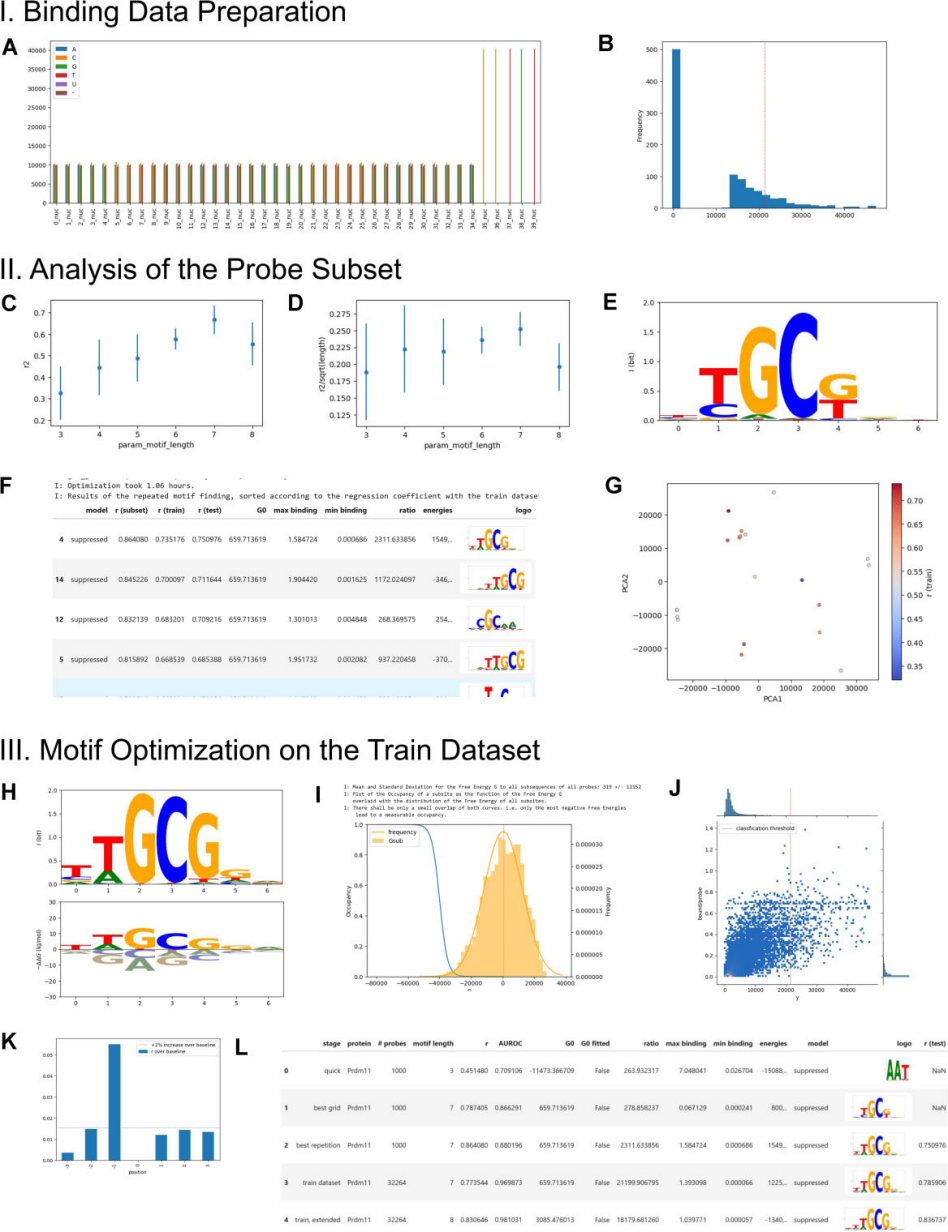
Explorative motif analysis with a Jupyter notebook. Depiction of the main steps of the workflow (see material and methods) for the transcription factor PRDM11. (**A**) visualization of the composition of the probe sequences used for analysis; here the input sequences were padded with five nucleotides of the constant probe part. (**B**) Intensity distribution of the probe subset consisting of 500 low-intensity probes and 500 high-intensity probes. The red vertical line is the classification threshold calculated from the complete dataset as mean +4 standard deviations. (C/D) GridCV search to find the motif length. The core motif length is chosen from the maximal of *r*^2^/sqrt(motif length) visualized in (D), here 7. (**E**) Logo of the best model from the GridCV search. (**F**) Repeated optimization with the core motif length; only top hits are shown. (**G**) Corresponding PCA of the energy matrix of the found motifs. Red dots have the highest regression quality. (**H**) The best motif from list F is chosen as the starting condition for optimization using the data from the complete train dataset. Shown are the logo and energy logo presentation of the obtained energy matrix. (**I**) Distribution of the binding energies over all subsequences of motif length and all probes (histogram with fitted normal distribution) The vertical line is Δ*G*_0_, the free energy of unspecific binding. Only a few binding events have sufficient (negative) free energies to lead to measurable subsequence occupancy (blue line). (**J**) Heatmap of binding data versus predicted probe occupancy. The large excess of low binding probes is visualized by colour (see the red dot close to the origin) and the bordering histograms. The red line is the classification threshold. (**K**) Investigations of the bordering positions relative to the core motif (H). Here, one position 5′ to the core motif was included in the next analysis. (**L**) Summary of the motif models analysed during the workflow. The first line is a quick optimisation to test for data integrity. The workflow also provides additional figures to assess the classification performance (not shown).

The input data typically amounts to several ten thousand probes. To speed up the calculation of the motif, the train data is further downsampled for initial calculations. Typically, the probes with the 4% highest and the lowest 4% input binding signal are taken and further down sampled to a maximum of 1000 probes (Figure 1B). This subset contains sufficient probes to find a motif present in the high-rank probes versus the background probes devoid of this motif.

This subset is used to estimate the length of the motif. To achieve this, a five cross-validation is performed on the subset in a sensible range of motif lengths. The average regression coefficient of the cross-validations of a given motif length is then used to define the length of the core motif. Typically, the length of the core motif can be chosen as the optimum from *r*^2^ divided by the square root of the motif length (Figure 1C, D).

A typical problem in optimization, especially for functions with many parameters is the lack of convergence to find the global minimum. Therefore, the motif finding is repeated 20 times on the subset to sample the energy landscape better. In the standard workflow, 20 local optimization are initiated with a random start motif. The motifs detected by these replicate optimizations are then compared with a 2D principal component analysis (Figure 1G). Typically, in the PCA visualisation, a cluster of motifs with high-ranking regression coefficients is found. In case the probes are double-stranded, it is to be expected that two clusters form with motifs that are reverse complementary to each other. The presence of these two corresponding clusters is an internal control for the validity of the optimization.

Complementary to the PCA analysis, the 20 independently found motifs with their corresponding energy matrix are then used to calculate the regression coefficient on the full training set and the test dataset (Figure 1F). The motif with the best regression coefficient on the training set is then carried on for the optimization.

The local optimization of the complete training dataset is started with the best energy matrix previously detected in the subset. The subset was balanced between low- and high-binding probes. In contrast, the training dataset contains much more low-binding probes. This leads to a readjustment of the motif to avoid spurious binding to the very large number of low-binding probes. Nevertheless, the strong signals of the motif present in the high binders of the subset are maintained.

The optimized motif on the training set still has the length found to be optimal during the initial analysis of the subset with the enriched fraction of high-binding probes. With the complete training dataset, the optimization is challenged with a higher number of probes including an increased fraction of background probes. Thus, it is highly probable that the regression is improved when neighbouring positions to the core motif are also considered since the higher motif lengths entail a better discriminatory power of the motif.

Therefore, the next step in the workflow is to investigate whether the extension on the 5′ or 3′ side of the core motif by 1 to 3 positions improves the motif as assessed by the regression coefficient (Figure 1K). For computational efficiency, the energy matrix of the core motif is held constant, whereas each neighbouring position is independently optimized starting from a zero energy matrix. In the standard workflow, an increase of the regression coefficient by 2% is deemed to include the respective position for further analysis. Based on this decision, a new starting energy matrix is generated with the core motif energy matrix and the singly optimized energy matrices of the added neighbouring positions. This new extended motif is then optimized with the training dataset.

In the next step, the motif is further extended by one bordering position at both ends, but only if the information content of the respective current 5′ and 3′ positions exceeds 0.25 bit. In a final optimization step, the obtained motif (including the extended and bordering positions) is challenged. The contribution of all positions in the motif is evaluated by setting the respective energy matrix of this position to zero and by determining the Pearson regression coefficient of the modified energy matrix. Relevant positions show a clear decrease in regression quality. In the current implementation, a change of regression coefficient above −2% is deemed acceptable to drop the respective position. Positions can only be dropped if they are continuous positions at the border of the motif. A final optimization is then carried out, starting with the energy matrix devoid of the irrelevant bordering positions.

In the standard implementation the unspecific free energy of binding $\Delta {{G}_0}$ is estimated based on motif length and the typical energy values in the matrices of nucleic acid binding proteins. If the reported occupancies of the optimization indicate saturation of the PBM (many probes exceeding an occupancy of 1) or very low occupancy in all probes, it is advisable to adjust $\Delta {{G}_0}$. This can be done in two ways: by including $\Delta {{G}_0}$ in the fit (targeting to a maximal user-defined occupancy, usually 1) or by finding an appropriate $\Delta {{G}_0}$ by holding constant the energy matrix while monitoring maximal occupancy and the quality of linear regression. These analyses are reported for the information of the user. In most cases, an adjustment is not necessary.

Software and data used is available at https://github.com/l-i-g/Motiffinder and https://dx.doi.org/10.5281/zenodo.10560171.

## Results

In a first step, the scikit-learn regressor for motif identification embedded in the Jupyter notebook was validated with published PBM and RNAcompete datasets.

The majority of the PBM datasets analyse the specificity of eukaryotic transcription factors and use double-stranded DNA as probes (1). Four transcription factors were selected from the DREAM5 challenge (3). As can be seen in Table 1 the Motiffinder regressor found the same motifs as BEEML-PBM (23,24) at a comparable regression quality. Only for the transcription factor Sox10 the regression quality was significantly poorer, although the same motif was detected. A possible reason could be the lower overall signal intensity of this particular dataset. Although the same protein microarrays were used for the four transcription factors reported in Table 1, on the SOX10 array only very few high-binding probes were detected (Supplementary Figure S1). In addition, the workflow used by Motiffinder does not filter and preprocess the fluorescence data.

**Table 1.** Motifs detected by the Motiffinder on selected datasets of the Dream5 challenge

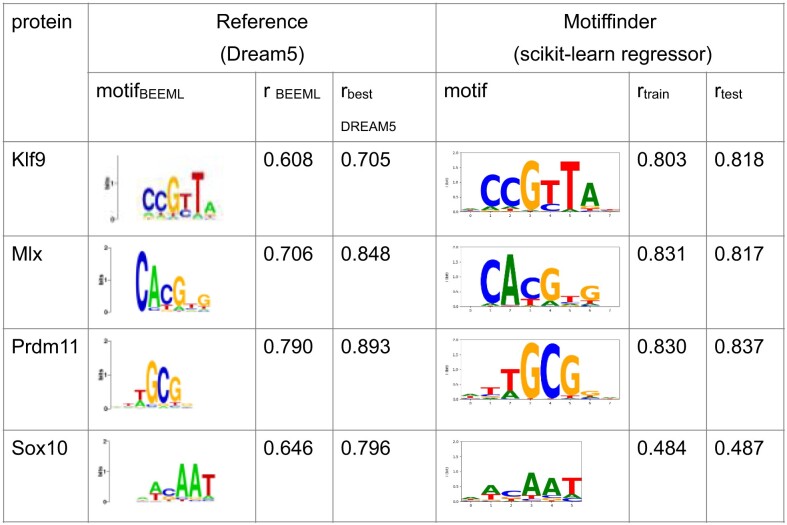

Shown are the logos obtained with BEEML-PBM along with the correlation coefficient obtained with BEEML-PBM and with the best-performing algorithm of the respective transcription factor. The motifs detected by Motiffinder are very similar, and the Pearson correlation coefficients are comparable or better, except in the case of Sox10.

Next, RNAcompete datasets were selected to test the performance of the Motiffinder on single-stranded nucleic acids. The RNAcompete datasets were analysed by the original authors (11,10,25) with the Seed-And-Wobble algorithms (2). This algorithm is based on the overrepresentation of motifs in the high-binding probe sequences. It is known that this algorithm tends to construct a motif with a too high information content (3). Indeed, the motifs obtained by Motiffinder generally had a lower information content than the motifs obtained by RNAcompete (Table 2). In particular, for the proteins PTB1 (Polypyrimidine tract-binding protein 1) and HNRNPA1 (Heterogeneous nuclear ribonucleoprotein A1) the motif detected by the Motiffinder is only a dinucleotide. Nevertheless, these low-information content motifs agree well with the motifs found by the DeepBind algorithm. In all cases the regression quality of Motiffinder was significantly better than that of RNAcompete and approached in quality the much more complex DeepBind algorithm (26).

**Table 2.** Motifs detected by the Motiffinder on selected RNAcompete datasets

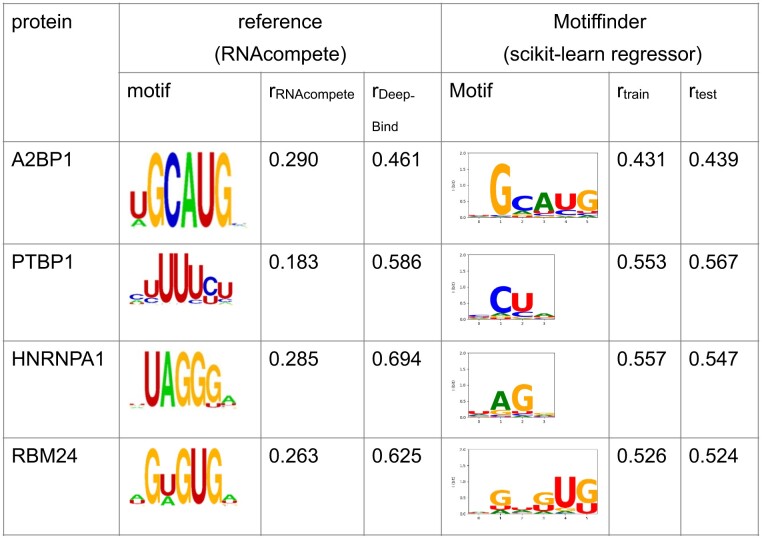

The logos (https://hugheslab.ccbr.utoronto.ca/supplementary-data/RNAcompete_eukarya/Experiment_reports/RNAcompete_report_index.html) as well as the Pearson correlation coefficient obtained with RNAcompete and DeepBind are taken from the Supplementary Table S2 from Alipanahi *et al.* (26).

In summary, the comparison with known motifs confirmed that the Motiffinder algorithm can detect motifs in binding experiments with single- as well as double-stranded nucleic acid sequences.

After having established that the Motiffinder regressor is able to find motifs from binding data based on a biophysical model, the sequence specificity of the T7 primase was determined by a reanalysis of the PBM experiments conducted by group of Akabayov (19,20). Noteworthy, the elucidation of the sequence-specific binding of a bacterial primase via a PBM experiment is much more challenging than the identification of the sequence specificity of a typical transcription factor: Firstly, the binding of the primase to the template is weaker, and secondly, the specificity of the binding is much less pronounced compared to transcription factors. Moreover, in the present case the binding experiments was carried out in the presence of ribonucleotides. This will allow the primase not only to bind to the template but also to move along the single-stranded template during an eventual priming reaction.

In a PBM experiment, probes are immobilized on the microarray surface. Then the protein of interest is incubated with the microarray slide. The protein will bind to the probes according to its preference for certain sequences. In the case of proteins that bind with low sequence specificity, binding events will occur with the majority of the immobilized probes. To be more specific, a protein with a sequence specificity of 4 bit (that is the unambiguous recognition of 2 bases) will bind to a random nucleic acid sequence in average every 16 bases. The probes of a standard PBM have a variable sequence of 35 nucleotides. Since typical PBM experiments only allows to identify the probe, which is bound by the protein but not the position where the protein binds, the uncovering of the sequence specificity of low-specificity binding proteins is more difficult, especially in the presence of experimental noise.

It was therefore tested beforehand whether a known motif could be recovered from simulated binding data to which noise was added to a varying degree. The analysis was based on 3149 probe sequences of the T7 DNA binding data from the group of Akabayov (20). 100 random motifs with a length 3 bases were generated with an information content between ∼1 and ∼5 bit. For each random motif, the occupancy on the 3149 probes was calculated based on the thermodynamic model. The unspecific binding energy $\Delta {{G}_0}$ was adjusted so that no oversaturation of the probes occurred, i.e. a maximal occupancy of 1.2 proteins per probe was targeted.

The noise in the T7 primase dataset was estimated using the replicate measurement of the probes (20). In the simulation, we added a single noise term (‘weak’) or two or four times of noise (‘medium’ and ‘strong’). The simulated binding data (with and without added noise) from each random motif then went through the Motiffinder algorithm to find the motif which best explained the simulated binding data. For each random motif, 10 optimization trials were performed, and the motif with the best regression coefficient between predicted occupancy and simulated/noised occupancy was kept for further analysis.

**Figure 2.**
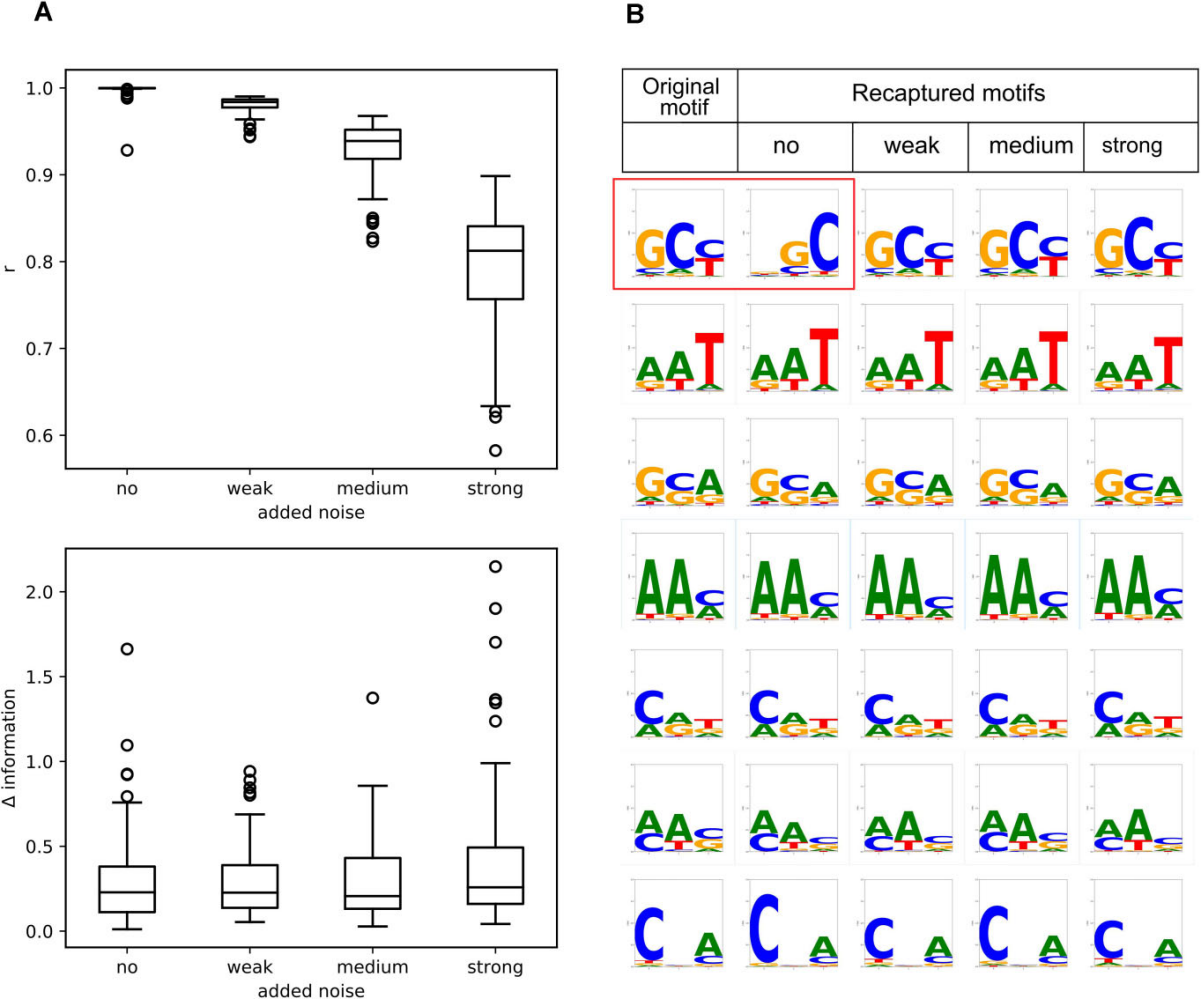
Quality of the motifs recovered with simulated and noised binding data. Probe occupancies were calculated for 100 random motifs (1–5 bit information content), and noise was added to the binding data. (**A**) Plotted are the distributions of the Pearson regression coefficients between predicted occupancy and the simulated and noised binding data for all 100 random motifs. The regression quality decreases with the amount of noise added. However, the addition of noise only leads to a marginal change of the motif (Δ information) (**B**) Logo representations of the original random motif and the recaptured motif calculated by the Motiffinder algorithm after the addition of noise. Shown are the last seven random motifs. In most cases, the motif could be recovered very well. One example of a high information difference and poor regression quality is seen in the top row (boxed in red) between the logo of the original motif and the logo obtained with simulated binding data and no noise added. In this case, the information difference was high (1.7 bit) since the motifs are shifted by one position. Most motifs are, however, very well recaptured.

As expected, the addition of noise has a clear impact on the regression coefficients obtained from the Motiffinder algorithm (Figure 2A). In most cases with no noise added, the algorithm found exactly the energy matrix of the random motif that was used to generate the binding data; therefore, the regression coefficients were close to one. In a single case, a regression coefficient of ∼0.93 was detected. In this case the detected motif was shifted by one position relative to the random motif (see Figure 2B, red box). The addition of noise progressively leads to a decrease in regression coefficients (Figure 2A).

Interestingly, however, when the motifs found by the Motiffinder algorithm under the conditions of varying amounts of noise are compared, it is found that the sequence logos are nearly identical (Figure 2B). We quantified the motif resemblance by calculating the root over the sum of all squared differences in the information contribution of each base and position. With this metric, which reflects the visual resemblance of information logos, we find that the addition of noise has only a limited effect on the motif which is recaptured from the noised data (Figure 2B). Thus, the simulation demonstrates that even noised binding data is sufficient to recover the correct motifs, even if the motifs have a low information content between 1 and 5 bits.

After having verified that the probes of the custom-made PBM allow for the detection of low-information motifs in noised PBM data, the binding data was reanalysed with the thermodynamic model incorporated in the Motiffinder algorithm.

The T7 custom PBM array used by Soffer et al. has a central 5′-GTC motif (the known priming site of the T7 primase) flanked by variable sequences: 17 nucleotides on the 5′ side and 16 nucleotides on the 3′ side. The probes also consist of 24 nucleotides further on the 3′ side, which are invariant. The constant sequence contains an additional 5′-GTC motif at its border facing the variable sequences. The purpose of the constant sequence is to link the variable part of the probe to the surface. Nevertheless, it can be assumed that this part of the probe is also accessible for protein binding. We therefore conducted the analysis in two different settings. One with probe sequences devoid of the 24 constant nucleotides (‘no padding’) and one with adding 6 nucleotides of the constant sequence (5′-GTCTTG, ‘padding'). The dataset contains the median of replicate measurements from 3149 probe sequences, normalized between 0 and 1, and square-rooted (20). The raw data was therefore squared to approach a linear relationship between the measured fluorescence signal and the occupancy.

**Figure 3.**
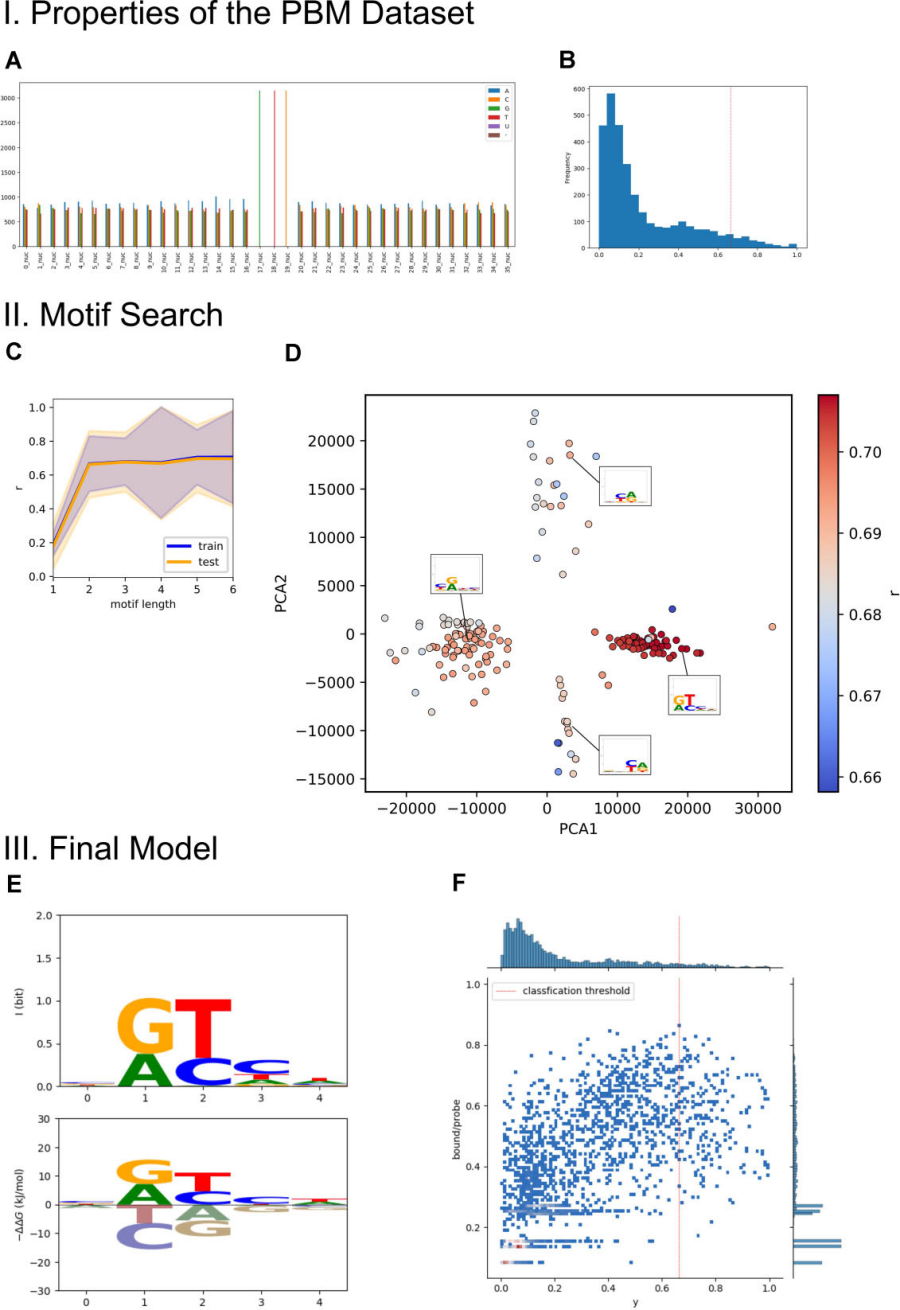
Explorative motif analysis of the T7 primase dataset. (**A**) Visualization of the composition of the unpadded probe sequences used for analysis. The custom-made PBM contained a central GTC motif in all probe sequences. (**B**) Histogram of the normalized binding scores. (**C**) Validation curve with mean and standard deviation of Pearson r. Means and individual test and train scores were nearly identical in a 10-fold repeated 5-fold cross validation with the motif length as parameter. (**D**) Motif finding was repeated 200 times and the obtained energy matrices were investigated via Principal Component Analysis. Four clusters with a representative logo are indicated. The colour of the dots indicate the quality of the underlying regression coefficient of the motif. PCA1 and PCA2 explained 62% and 17% of variance, respectively. (**E**) Logo and Energy logo of the final motif with five positions. F: Heatmap and histograms of predicted probe occupancies (bound/probe) vs. normalized measured binding score (y). The regression coefficient was 0.723.

Compared to the standard workflow described above, the Jupyter workflow was slightly adjusted for the T7 primase binding data. Firstly, the thermodynamic model requires an estimation of $\Delta {{G}_0}$. In case of the T7 primase PBM data, the Motiffinder algorithm estimated a too strong unspecific affinity of the T7 primase to the probes. This leads to an oversaturation (> 1 bound protein/probe) for a number of probes. Probe oversaturation is not possible from a biochemical viewpoint and therefore needs to be prevented by the algorithm. We therefore included $\Delta {{G}_0}$ in the optimization and penalized the maximal occupancy over all probes deviating from 1. Secondly, as the number of probes is low, there is no need to construct a subset of the training dataset for initial motif discovery. Instead, the complete dataset (3149 sequences) was directly used. A 5-fold cross-validation was repeated 10 times varying the parameter motif-length. The training and test scores run parallel indicating that the underlying models did not overfit (Figure 3C). Further, the motif length of 2 already achieved a good regression with a mean of ∼0.65 which was only marginally improved up to a motif length of 6 with a mean of ∼0.68. The motif space was then extensively sampled with 200 local optimizations of the motif length of 4 nucleotides and yielded roughly four clusters of motifs in a 2D PCA (Figure 3D). In both settings, with and without padding, the cluster with the highest regression coefficient was similar to the know 5′-GTC priming motif. The motif with 4 positions had already a high regression quality, however extending the motif by one position on the 5′ side even improved the model further to *r* = 0.723 (Figure 3E and Table 3). The final regression quality is in the range of the regression quality obtained with the transcription factors. The mean absolute error (mae) is close to the values found by Soffer *et al.* (0.084–0.112) through a more difficult-to-rationalize machine learning model (20). The identified motif in both settings agrees well with the known priming specificity (5′-GTC) reflecting a potential link between binding and priming specificity.

**Table 3.** Analysis of the T7 primase binding data

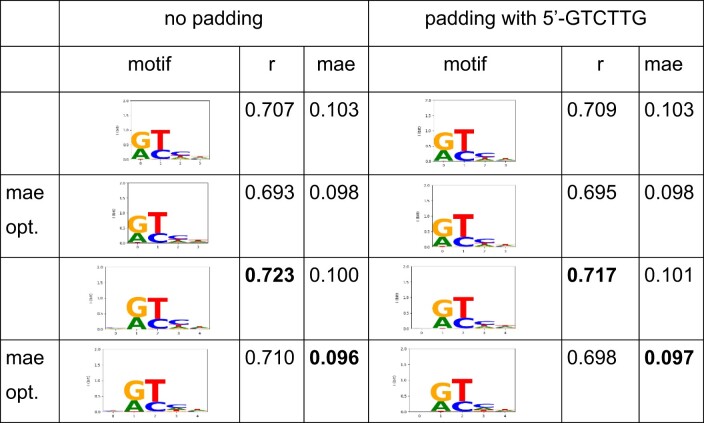

The second variant (‘padding with 5′-GTCTTG’) included the part of the constant region that contained a second 5′-GTC priming site of the T7 primase. Both analyses yielded comparable results with a four to five nucleotide-long motif with two dominant positions. For both variants, the models were fitted to maximize r and then refitted to minimize the mean absolute error (row: mae opt.).

## Discussion

In this work, the reanalysis of the PBM binding data of the T7 primase was used to develop a generic regressor which can identify motifs from large scale binding data. The regressor realised in the scikit-learn environment along with the Jupyter notebooks allow a relatively easy, explorative, and flexible analysis of nucleic acid binding data.

The prime intention of this work was however to elucidate the binding specificity of the T7 primase. This primase is a well-studied primase and can serve as a model to develop experimental approaches to uncover the binding specificity of primases or more generally, of nucleic acids binding proteins with low sequence specificity.

Concerning sequence specificity for primases, two different levels of specificity should be distinguished: The primases may bind preferentially to certain sequences (binding specificity), and they may initiate and complete primer synthesis only at certain sites (priming specificity). The priming specificity of some bacterial primases is well investigated, and typically priming starts at a specific trinucleotide sequence in the template (13). Possibly, the primase also binds preferentially at these priming sites. However, it is more likely that the binding specificity is more relaxed than the priming specificity. A suitable template sequence might be bound, but for dinucleotide synthesis to occur, additional conditions might need to be met. This could include correct positioning of the initiating and elongating nucleotide, base pairing of both nucleotides with the template, and additional interactions with the template. In this context, it should be stressed that the primase is one of the few enzymes where the interactions with three substrates needs to be coordinated by the enzyme. The catalytic step may only occur efficiently under optimal steric conditions of the precatalytic complex. Studies of the bacterial RNA polymerase (which catalyses a similar reaction) have revealed that the initiating nucleotide (which becomes the 5′ end of the transcript) stacks with a purine base in the template strand (27). A similar stabilization of the precatalytic complex is seen in archaeoeukaryotic primases. Here, pi-pi interactions between the protein (a tyrosine side chain), both nucleotides, and an adenine base in the template bind and orient the nucleotides (28). Thus, in both examples, the contribution of the template restricts productive priming beyond the binding specificity of the protein alone.

The priming specificity of the T7 primase is usually being described with the trinucleotide sequence 5′-GTC. The third base is cryptic, meaning that the primer end pppApC base pairs with the first two nucleotides of the priming site (5′-GT) thus the cytosine itself is not a templated nucleotide. In the context of the replisome the task of the T7 primase is the repeated synthesis of a short ribonucleotide primers on the lagging strand which is later extended to the Okazaki fragments. The T7 primase has a strong preference for CTP over the other ribonucleotides for the extension reaction (29). At the moving replication fork T7 primase synthesizes a four ribonucleotide long primer which is then transferred to the T7 DNA polymerase for further elongation with dNTPs. The specificity for the trinucleotide 5′-GTC together with the strong extension preference towards CTP therefore leads to the effective priming specificity with 5′-GG**GTC** being the best priming site followed by 5′-TG**GTC** and 5′-GT**GTC** (18,30).

The priming specificity can be rather easily determined as it materialize in the primer product. The binding specificity, however, is more difficult to measure as even short templates present a high number of potential binding sites (subsequences of the template DNA) of different sequences. These circumstances complicate a straightforward determination of the binding specificity of proteins with only short motifs. Instead, it is required to measure the affinities to a high number of probe sequences and then to extract the model the binding specificity as done by previous studies (19,20) and in this work.

The group of Akabayov analysed the binding specificity of the T7 primase with a custom-made protein binding microarray in two studies. In their first study, they observed that the sequence context around the central 5′-GTC motif on the probes of the PBM influences the binding strength (19). Sequence flanks with a T/G composition are bound better by the primase than sequence flanks of A/G. The latter templates also lead to longer primers with T7 primase alone but not with the T7 holoenzyme composed of a primase and a helicase domain. In view of the results presented in this reanalysis the better binding to the flanks of T/G composition can be explained by the better agreement of 5′-GT dinucleotides with the identified motif 5′-purine-pyrimidine as opposed to the A/G flank consisting only of purines. The better binding is caused by the additional weak binding of the T7 primase to the flanks.

In the second study, the binding specificity was investigated more closely with a dataset comprising 3149 probe sequences (20). They tried different methods for feature extraction from the probe sequences and different machine learning algorithms to model the binding data from the PBM dataset. Their final model used the *K*-mers frequency as features and LASSO linear regression. The model was fivefold cross-validated and achieved a regression quality between the mean absolute error of 0.171 (K = 1, equivalent to the nucleotide frequencies) and 0.084 (*K* = 4, frequencies of tetramers). It should be noted that this procedure can produce solutions which might present different ‘motifs’ in the different folds of the cross-validation. A Pearson regression coefficient was not reported, and the regression between binding score and predicted binding score was not plotted either.

Machine learning has its merits in being able to analyse complex datasets, but it also has the disadvantage delivering models that are poorly explainable. The low explainability is caused by the complex structure of the models and their high number of parameters. To avoid overfitting, the models therefore need to be carefully validated.

The complexity of the present dataset is not overly high, and well-established procedures for analysing PBM dataset have already been developed (3). The present reanalysis of the binding data with a strictly biophysical model achieved an overall comparable regression quality and delivered an easily understandable result, which is the base- and position-specific energy matrix (Table 3). The energy matrix can be visualized as energy or information logo and is immediately understandable to biochemists and molecular biologists.

The explorative data analysis using the Motiffinder regressor also revealed that the motif length has only a limited impact on the regression quality. A motif length of 2 already achieves a Pearson *r* ∼ 0.67 (Figure 3C) and longer motif lengths only marginally improve the regression quality to *r* ∼ 0.72 (Table 3 and Figure 3C). The importance of two positions is clearly visible in the logos of the found motif.

The information content of the motif is rather low, with only ∼2.5 bits. Thus, a motif is present on average every 2^2.5^ (∼6) nucleotides. In a recent characterisation of the priming specificity of the archaeoeukaryotic primase from *Nanoarchaeum equitans*, the priming specificity was found to be approximately 3 bits and the binding specificity can be expected to be even lower (31) underscoring the difficulties in determining the primase binding specificity in contrast to the priming specificity.

The binding motifs of the T7 primase identified with the Motiffinder agree with the known priming specificity. This means that the weak preferential binding could support the initiation of primer synthesis at the preferred priming site 5′-GG**GTC**. There is no structural information on the precatalytic complex of template and nucleotides bound by a bacterial primase. The cryo-EM structures of the replisomes of bacteriophage T4 and bacteriophage T7 show that the template and primer/template is bound by the zinc-finger domain (ZFD) and by the RNA polymerase domain (RPD) (32,33). In both cases, the 5′ primer end is bound by the ZFD, while the 3′ end is closer to the active site of the RBD. However, since the primers had a length of 5 and 6 nucleotides, respectively, the conformation is different than can be expected for the precatalytic primase complex with only the elongating and initiating nucleotide in place. Nevertheless, previous biochemical investigations confirmed that template and nucleotides are bound cooperatively with involvement of both domains. Early studies already showed that the dissociation constant for the T7 primase towards template is in the micromolar range and that the affinity towards the template increases in the presence of nucleotides (16,34). As CTP is the preferred nucleotide in the elongating nucleotide pocket of the primase (see above) it was concluded that the CTP bound by the primase could help in selecting the priming site (34). Moreover, the zinc finger domain of the primase is responsible for specificity towards the cryptic base cytosine in the priming site motif 5′-GTC (34). These findings were later substantiated with surface plasmon resonance experiments where 25 nucleotide long templates where immobilized and the binding by the T7 primase was measured (35). The binding of T7 primase increased greatly (*K*_D_ = 22 μM) when ATP and CTP, the two cognate nucleotides for the 5′-GTC priming site were present and the cryptic cytosine was not omitted in the template sequence. Remarkably, the addition of only ATP or only CTP was not sufficient to increase the binding of the template. These finding reinforce the view that template binding involves both nucleotides and the zinc finger which recognizes the cryptic cytosine.

The PBM experiments were conducted in the presence of all four ribonucleotides. Consequently, the binding of probes will be influenced by the binding of nucleotides in the initiating and elongating nucleotide pocket located in the primase active site. In this work, we detected a binding motif with two dominant neighbouring positions. These two positions could reflect the two distinct nucleotide binding pockets that bind the initiating nucleotide and the elongating nucleotide. The primase activity of the T7 primase suggest that CTP is preferred at the elongating site. The CTP could therefore base pair with the guanosine in the motif. A similar mechanism could apply for the initiating nucleotide. A preferential binding of ATP by the primase would explain the central thymidine in the dinucleotide motif. The motif detected by the Motiffinder is however ambiguous. This could be explained by the limited specificity for the CTP or the ATP at both nucleotide binding pockets or that size complementary between the bound nucleotide and the preferred template is already sufficient. In the latter scenario the bound CTP would allow a purine base and the bound ATP would allow a pyrimidine in the template.

In summary, the detected binding motif underpins that the observed binding specificity of the T7 primase is best explained by a dominant contribution of the template interacting with nucleotides bound in the active site of the T7 primase. In contrast, the much higher primase site specificity is due to additional steric and chemical constraints during dinucleotide synthesis and further extension to the tetraribonucleotide.

## Supplementary Material

gkae215_Supplemental_File

## Data Availability

The data underlying this article are available in Zenodo, at https://dx.doi.org/10.5281/zenodo.10560171. Please note that the PBM and RNAcompete datasets were generated by others. The original data can be also found under: https://hugheslab.ccbr.utoronto.ca/supplementary-data/DREAM5/, https://github.com/morrislab/RNAcompete, http://hugheslab.ccbr.utoronto.ca/supplementary-data/RNAcompete_eukarya/, http://hugheslab.ccbr.utoronto.ca/supplementary-data/RNAcompete_eukarya/Experiment_reports/RNAcompete_report_index.html and https://github.com/csbarak/T7pdrs. The software is also available at: https://dx.doi.org/10.6084/m9.figshare.25062371.
