# Supplementary material for: Definition of the binding specificity of the T7 bacteriophage primase by analysis of a protein binding microarray using a thermodynamic model
Source: Nucleic Acids Res. 2024 Apr 10;52(9):4818–29. doi: 10.1093/nar/gkae215 (PMC11109968; doi:10.1093/nar/gkae215)
Supplement: gkae215_Supplemental_File [file gkae215_supplemental_file.pdf]

Supplement

A

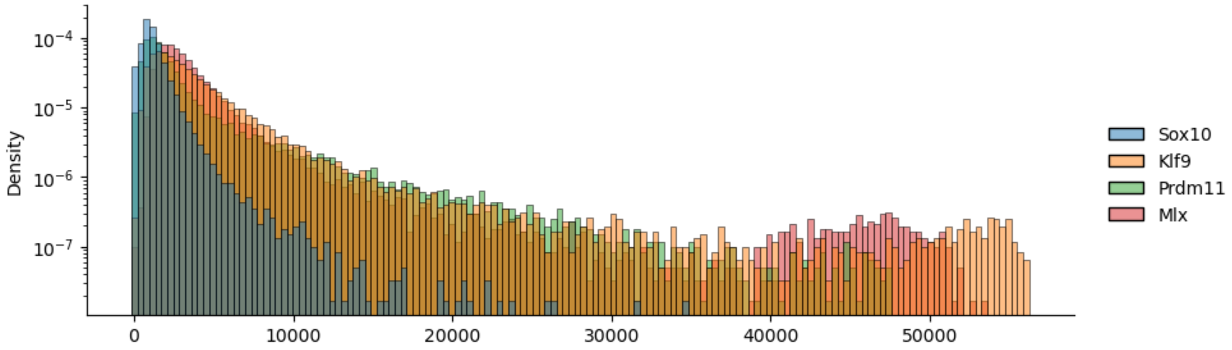

B

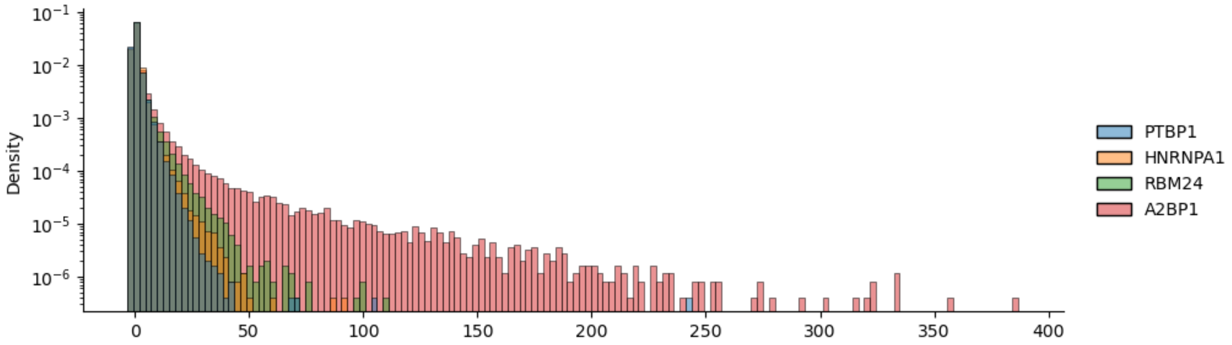

Supplementary Figure 1: Intensity distribution plots of the transcription factors (A) and the RNA binding proteins (B). In some binding experiments the fraction of probes with a high intensity was very low, e.g. SOX10 from the transcription factor and PTBP1 and HNRNPA1 among the RNA binding proteins.
